# Draft Genome Sequence of Textile Azo Dye-Decolorizing and -Degrading *Pseudomonas aeruginosa* Strain PFK10, Isolated from the Common Effluent Treatment Plant of the Ankleshwar Industrial Area of Gujarat, India

**DOI:** 10.1128/genomeA.00019-14

**Published:** 2014-02-06

**Authors:** P. R. Faldu, V. V. Kothari, C. R. Kothari, C. M. Rawal, K. K. Domadia, P. A. Patel, H. D. Bhimani, V. H. Raval, N. R. Parmar, N. M. Nathani, P. G. Koringa, C. G. Joshi, R. K. Kothari

**Affiliations:** a Department of Microbiology, Christ College, Rajkot, Gujarat, India; b Department of Biosciences, Saurashtra University, Rajkot, Gujarat, India; c Department of Biotechnology, Christ College, Rajkot, Gujarat, India; d Department of Animal Biotechnology, Anand Agricultural University, Anand, Gujarat, India

## Abstract

Here, we report the draft genome sequence of *Pseudomonas aeruginosa* strain PFK10, isolated from the common effluent treatment plant (CETP) of the Ankleshwar industrial area of Gujarat, India. The 6.04-Mb draft genome sequence of strain PFK10 provides information about the genes encoding enzymes that enable the strain to decolorize and degrade textile azo dye.

## GENOME ANNOUNCEMENT

Rapid industrialization has resulted in excessive environmental pollution, with one of the major sources being textile industry effluents. Synthetic dyes are used in textile, cosmetic, and food industries. These dyes are highly stable and difficult to degrade due to their complex aromatic structures (1). Azo dye accounts for almost 80% of commercial dyes all over the world per year, with a yearly production of >7 × 10^5^ tons (2). These colored industrial effluents are an obvious indicator of water pollution, and several physical-chemical methods have been used to eliminate them in wastewater (3, 4). However, these methods are expensive and produce large amounts of sludge. Consequently, microbial biodegradation techniques are considered a better alternative (5). Some microorganisms, including bacteria, fungi, and algae, are found to degrade a wide range of dyes (4, 6, 7). Various fungi have been reported to decolorize azo dyes using peroxidases or laccases (8). However, fungal treatment of colored industrial effluents is observed to be time-consuming (6).

*Pseudomonas aeruginosa* is reported to be a potential organism for the treatment of environmental pollution caused by textile industries (4). Whole-genome shotgun sequencing of *P. aeruginosa* strain PFK10, a potential textile azo dye-decolorizing and -degrading isolate from the common effluent treatment plant (CETP) of the Ankleshwar industrial area of Gujarat, India, was performed using the 318-Chip and 300-bp chemistry Ion Torrent PGM platform as per the manufacturer’s instructions. When the obtained sequence reads were subjected to reference-guided assembly against the whole-genome sequence of the organism *P. aeruginosa* M18 using GS Reference Mapper software version 2.3, the obtained draft genome of 6,041,907 bp showed the presence of 206 contigs of >200 bp in size.

The gene annotation was performed by submitting the sequences to the Rapid Annotations using Subsystems Technology (RAST) server (9). Annotation depicted the presence of 6,115 protein-coding sequences (CDSs), of which 3,095 CDSs were assigned to one of the 554 RAST subsystems. The genome contains 64 RNA molecules and 66.8% G+C content. The genome shows the presence of many genes coding for oxidoreductases, thus supporting the dye-decolorizing potential of the strain (10). The draft genome also showed the presence of laccase-like multicopper oxidases that have been reported in *Pseudomonas* spp. and found to decolorize various dyes by oxidation of the substrate coupled with reduction-releasing water (11).

Biochemical characterization showed that the isolate degrades various mono-azo, di-azo, tri-azo, and poly-azo textile dyes used in dyeing and printing industries. *P. aeruginosa* PFK10 secretes a fluorescent yellow-green pigment (pyoverdine). At the initial stage of growth, it forms biofilms, and at a later stage of growth, it produces a high amount of a polysaccharide substance. *In vitro* plate studies also showed that *P. aeruginosa* strain PFK10 hydrolyzes casein, starch, gelatin, glucose, fructose, mannitol, and citrate, and it produces important enzymes, like catalase and nitrate reductase. Information obtained from the whole-genome sequence about the metabolic pathways of the strain will help reveal the genes coding for enzymes involved in *P. aeruginosa* PFK10 that support its potential abilities to degrade textile azo dye, which can be used for the bioremediation of sites contaminated with textile effluents.

### Nucleotide sequence accession number.

The sequence of *P. aeruginosa* PFK10 has been deposited at GenBank under the accession no. AZBM00000000.
